# Fertility optimization in women with cancer: from preservation to contraception

**DOI:** 10.5935/1518-0557.20190011

**Published:** 2019

**Authors:** Anderson Sanches de Melo, Camilla Teles Vidal de Paula, Marcelo Augusto Feres Rufato, Mariana Carvalho Assad Carneiro Rufato, Jhenifer Kliemchen Rodrigues, Rui Alberto Ferriani, Jorge Barreto

**Affiliations:** 1 Fertility Center of Ribeirão Preto (CEFERP) - Ribeirão Preto - São Paulo - Brasil; 2 Member of Latin America Oncofertility Network, Oncofertility Consortium; 3 Centro Universitário Estácio – Ribeirão Preto – São Paulo – Brasil; 4 Department of Obstetrics and Gynecology, Ribeirão Preto Medical School, University of São Paulo. Ribeirão Preto – São Paulo – Brasil; 5 *In Vitro* Embriologia Clínica e Consultoria – Nova Lima – Minas Gerais – Brasil; Universidade Federal de Minas Gerais – Belo Horizonte – Minas Gerais - Brasil

**Keywords:** contraception, fertility preservation, embryo freezing, oocyte freezing, infertility

## Abstract

Advances in the early diagnosis and treatment of cancer have reduced mortality rates and improved patient survival. For this reason, professionals from different areas have strived to implement actions to increase patient quality-of-life during and after cancer treatment. Among these measures, integral attention in reproductive health is one of the main points for the inclusion, safety, and autonomy of female patients. The approach to fertility in these cases should include counseling on fertility preservation and contraceptive options. Oocyte/embryo freezing is an effective technique that does not delay the start of cancer treatment, since controlled ovarian stimulation can be initiated at any stage of the menstrual cycle. At the same time, contraceptive counseling should be conducted based on the eligibility criteria established by the World Health Organization and the Centers for Disease Control and Prevention. However, there is still a lack of studies on (i) the suitability of contraceptives to patients of reproductive age with relatively frequent tumors (lymphoma, leukemia, bone cancer), and (ii) the use of contraceptive concurrently with chemotherapeutic agents. Therefore, the choice of contraceptive method should consider other factors such as tumor type, thrombogenic risk factors linked to cancer/chemotherapy, immunosuppression, blood disorders (thrombocytopenia/anemia), bone mass reduction, metabolic/cardiovascular effects, and drug interaction.

## INTRODUCTION

Cancer ranks as the third cause of death in the world, with 1-2% of the cases of invasive cancer occurring during reproductive age (between 25 and 39 years). The most common types of cancer to affect young people are lymphoma, leukemia, germ cell (testicular) tumors, bone tumors, melanoma, and breast cancer, among other less frequent types (Aben *et al*., 2012; Desandes & Stark, 2016).

In the United States, the overall five-year survival rate for all invasive cancers among individuals aged of 15-39 years is about 82.5% (Keegan *et al*., 2016). Despite the increased incidence of tumors, these numbers show that advances in cancer early diagnosis and treatment have reduced mortality rates and improved the global survival of cancer patients (National Cancer Institute, 2013). Therefore, professionals from different areas have been working to improve the quality-of-life of patients during and after cancer treatment (Loren *et al*., 2013; Peccatori *et al*., 2013; The Practice Committee of American Society for Reproductive Medicine, 2013). One of the main points embedded in the measures taken to safeguard the inclusion, safety, and autonomy of individuals surviving cancer is integral attention to reproductive health.

The potential gonadal toxicity of radiotherapy and some chemotherapeutic agents is a relevant factor that may interfere with the quality-of-life of cancer survivors. While some types of cancer treatment cause irreversible infertility, others may result in a temporary loss of reproductive function. In other cases, treatment may have little impact on fertility. Unplanned pregnancy during cancer treatment may alter the prognosis and the evolution of pregnancy (maternal cardiac failure, miscarriage, low birth weight, and preterm birth) (Pentheroudakis *et al*., 2010).

A holistic approach to fertility for cancer patients should include two key points: preserving fertility and guiding effective contraception prior to the initiation of cancer treatment. Despite the importance of an integral approach to fertility for women set to undergo cancer treatment, the practice still faces barriers. They revolve around the lack of appropriate recommendations for the preservation of fertility, concerns about possible delays to initiate cancer treatment, overestimation of assisted reproduction costs, lack of knowledge about fertility preservation/contraception options, lack of reference centers for integral health care, and disregard for fertility in relation to the severity of the disease, to name a few (Levine *et al*., 2010). Most notably, the clinical condition of the patient and the prognosis entailed by the disease are relevant in the decision to advise women with cancer to seek fertility preservation (Schüring *et al*., 2018).

Some medical societies are working intensively to raise awareness of physicians, nurses, multiprofessional staff, and patients in order to increase the access of cancer patients to fertility preservation (Loren *et al*., 2013; Peccatori *et al*., 2013; The Practice Committee of American Society for Reproductive Medicine, 2013; Ataman *et al*., 2016). However, there are no effective policies on contraceptive counseling for women set to undergo cancer treatment. In practice, guidance on fertility is fraught with gaps. Cancer patients with access to fertility preservation may maintain adequate reproductive function and the ability to become pregnant during treatment. Lack of information on the maintenance of reproductive function during cancer treatment and unawareness of the benefits of gamete storage may surprise patients with an unplanned pregnancy, unless proper contraceptive advice is offered.

The World Health Organization (WHO) and the Centers for Disease Control and Prevention (CDC) looked into the possible effects of contraceptives in cases of cervical, breast, endometrial, ovarian, and liver cancer, and included these conditions in their medical eligibility criteria (World Health Organization, 2015; Curtis *et al*., 2016). Although leukemia, lymphoma, and bone tumors are relatively frequent in individuals of reproductive age, there is no guidance on the use of contraceptives for these conditions. In addition, there is no reference to contraceptive eligibility and the possible interactions they may have with radiotherapy and chemotherapeutic agents often used in women of reproductive age. These issues further limit the access to contraceptives at this important stage of cancer management, warranting the need for protocols to improve the approach to fertility for cancer patients of reproductive age.

In general, prescribing effective contraceptives to women with cancer may yield benefits such as decreasing unplanned pregnancy rates, preventing changes to the prognosis of cancer and/or pregnancy, introducing non-contraceptive benefits [irregular menstrual cycles might worsen quality-of-life and even change cancer prognosis (e.g. leukemia)], improving sexual satisfaction by avoiding unplanned pregnancies, optimizing quality-of-life, and respecting women’s autonomy in relation to their fertility.

**Figure 1:**
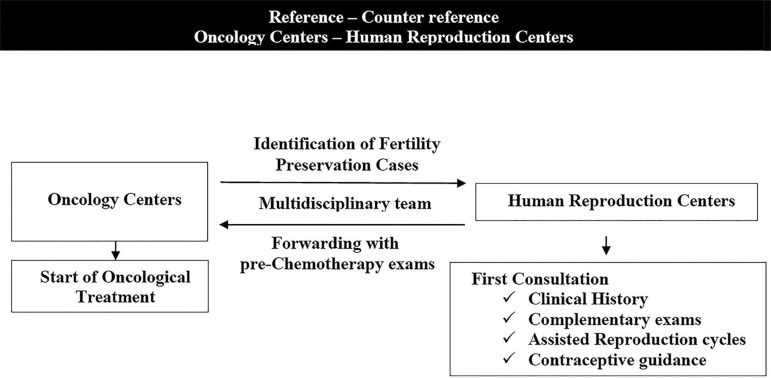
Holistic approach to fertility for women with cancer This figure shows a practical multidisciplinary flowchart covering the approach to fertility for women with cancer, considering the preservation of fertility and the prescription of effective contraception. Although contraception guidance can be performed in any setting by any physician, the appropriate place for this to occur is a human reproduction center, after the completion of fertility preservation procedures and prior to the initiation of cancer treatment. Interdisciplinary communication with agility and the availability of complementary tests are fundamental for an integral approach to the reproductive health of cancer carriers. This system does not delay the start of cancer treatment, while carrying out tests before chemotherapy optimizes reference and counter-reference.

Although contraceptive counseling can be performed in any setting by any physician (World Health Organization, 2015; Curtis *et al*., 2016), cancer patients should be provided counseling immediately after they have undergone a procedure for fertility preservation in a human reproduction center. This practice would provide safety to patients, oncologists, and gynecologists, and would preserve the autonomy and inclusion of patients in their reproductive planning process. Interdisciplinary communication, prompt availability of complementary tests for fertility optimization, and timely initiation of cancer treatment are all required (Figure 1). This study aimed to design a practical flowchart for a holistic approach to fertility for women with cancer, including preservation of fertility and contraception.

## FERTILITY PRESERVATION

### Counseling

Fertility preservation for women with cancer is an assisted reproductive technology (ART) treatment that enables the generation of a child with genetic inheritance after an adequate disease-free time interval. Many cancer therapies have a high risk of gonadal toxicity and may permanently deplete one’s reproductive potential. The ensuing infertility can bring irreparable emotional/social repercussions that disrupt the autonomy and desire of the individual of having a family (Loren *et al*., 2013; Peccatori *et al*., 2013; The Practice Committee of American Society for Reproductive Medicine, 2013).

**Figure 2:**
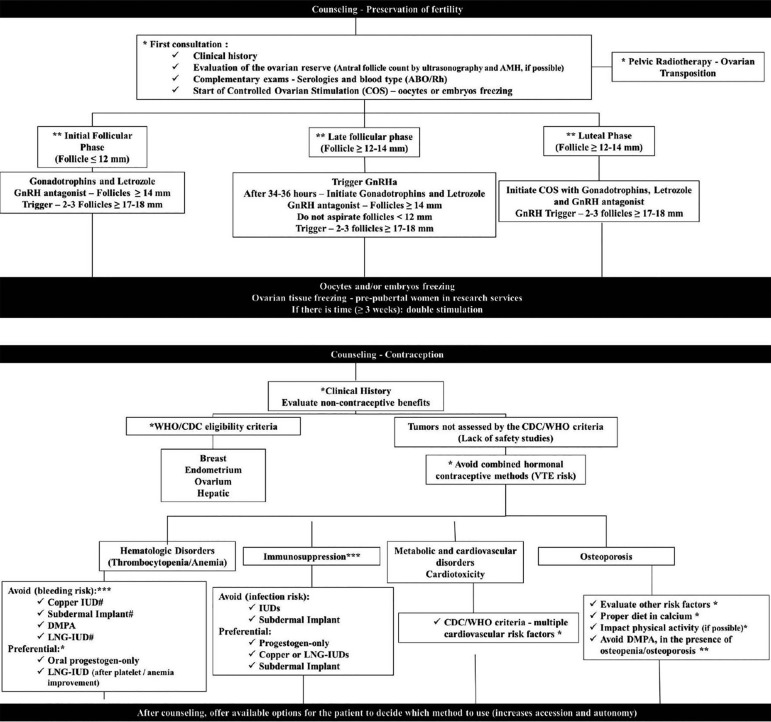
Practical flowchart to discuss fertility preservation and contraception with women with cancer *recommendation level A; **recommendation level B; ***recommendation level C; # LNG-IUD and Cu-IUD should not be implanted, but can be kept in place if the patient had already been using an IUD. (GnRH: Gonadotropin-releasing hormone; AMH: Anti-Müllerian Hormone; WHO: World Health Organization; CDC: Centers for Disease Control and Prevention; VTE: venous thromboembolism; IUD: intra uterine device; DMPA: depot medroxyprogesterone acetate; LNG-IUD: levonorgestrel-releasing intrauterine device)

Before beginning an ART cycle for fertility preservation, gathering the patient’s clinical history in detail and assessing her ovarian reserve may help the counseling process and increase her chances of becoming pregnant in the future. Information such as age, previous attempts to get pregnant, characterization of the menstrual cycle, current and previous diseases, medications in use, antral follicle count on ultrasound examination, and levels of follicle stimulating hormone (FSH) or anti-Müllerian hormone (AMH), when possible, are useful. Altogether, these data are used to advise the patient on her reproductive potential and possible success of the fertility preservation procedure (Figure 2) (Loren *et al*., 2013; Peccatori *et al*., 2013; The Practice Committee of American Society for Reproductive Medicine, 2013).

In 2013, the American Society of Clinical Oncology (ASCO) established a practical guide to optimize fertility preservation in cancer patients. The purpose of this protocol was to warn healthcare workers (oncologists, radiation therapists, gynecologic oncologists, urologists, hematologists, pediatric oncologists, surgeons, nurses, social workers, psychologists, and other non-medical workers) of the need to discuss the chances of infertility and the options to preserve fertility before cancer treatment with their patients. Patients interested in preservation methods should be referred to human reproduction clinics after having their prescribed chemotherapy or radiotherapy regimen assessed for gonadal toxicity (Loren *et al*., 2013).

Counseling on the fertility impact of cancer treatment should include the potential gonadal toxicity of chemo and radiotherapy. The European Society for Medical Oncology (ESMO) and the ASCO published a list of the possible effects of gonadal toxicity from several chemo and radiotherapy regimens (Table 1) with the aim of assisting in the decision to undergo preservation of fertility (Loren *et al*., 2013; Peccatori *et al*., 2013; The Practice Committee of American Society for Reproductive Medicine, 2013; Pentheroudakis *et al*., 2010). Another relevant bit of information in reproductive counseling is the data on live births per number of stored oocytes/embryos, so that patients are informed of the possible need to undergo more than one cycle of ovarian stimulation (Doyle *et al*., 2016).

**Table 1:** Potential gonadal toxicity according to the American Society of Clinical Oncology (ASCO) (adapted from Pentheroudakis et al., 2010; Lambertini et al., 2016).

Degree of risk	Type of Cancer Treatment
High risk (>80% risk of permanent amenorrhea)	HSC transplantation with cyclophosphamide/TBI or cyclophosphamide/busulfan External beam radiation to a field that includes the ovaries CMF, CEF, CAF, TAC x 6 cycles in women ≥ 40 years Melphalan Chlorambucil Dacarbazine Procarbazine Ifosfamide Thiotepa Nitrogen mustard
Intermediate risk (40 % - 60 % risk of permanent amenorrhea)	BEACOPP CMF, CEF, CAF, TAC x 6 cycles in women age 30–39 AC x 4 cycles in women≥40 years AC or EC x 4 → Taxanes Anthracyclines Cisplatin Carboplatina Ara-C (Cytarabine)
Low risk (<20 % risk of permanent amenorrhea)	ABVD in women ≥32 years CHOP x 4–6 cycles CVP AML therapy (anthracycline/cytarabine) ALL therapy (multi-agent) CMF, CEF, CAF, TAC x 6 cycles in women≤30 years AC x 4 cycles in women ≤40 years Bleomycin Actinomycin D Vinca alkaloids Mercaptopurine Etoposide Fludarabine
Very low or no risk (Risk of permanent amenorrhea)	ABVD in women <32 years Methotrexate Fluorouracil Vincristine Tamoxifen
Unknown risk (Risk of permanent amenorrhea)	Monoclonal antibodies (trastuzumab, bevacizumab, cetuximab) Tyrosine kinase inhibitors (erlotinib, imatinib) Taxanes Oxaliplatin Irinotecan

HSC: Hematopoietic stem cell; TBI: total body irradiation; CMF: cyclophosphamide, methotrexate, fluorouracil; CEF: cyclophosphamide, epirubicin, fluorouracil; CAF: cyclophosphamide, doxorubicin, fluorouracil; TAC: docetaxel, doxorubicin, cyclophosphamide; BEACOPP: doxorubicin, bleomycin, vincristine, etoposide, cyclophosphamide, procarbazine; AC: doxorubicin, cyclophosphamide; EC: epirubicin, cyclophosphamide; ABVD: doxorubicin, bleomycin, vinblastine, dacarbazine; CHOP: cyclophosphamide, doxorubicin, vincristine, prednisone; CVP: cyclophosphamide, vincristine, prednisone; AML: acute myeloid leukemia; ALL: acute lymphocytic leukemia.

### Fertility preservation techniques

**Figure 3:**
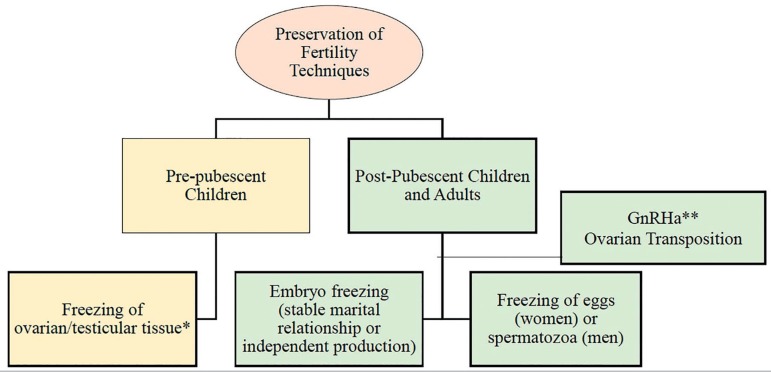
Fertility preservation techniques for cancer patients *Experimental approach **Not effective in fertility preservation Cryopreservation of embryos, oocytes, and spermatozoa are considered effective techniques for preserving fertility. Freezing ovarian and testicular tissue is experimental and should only be indicated in pre-pubescent children evaluated in reference services of research. The transposition of ovarian tissue is an option for people set to undergo pelvic radiotherapy. The use of the gonadotropin-releasing hormone agonists (GnRHa) is not effective for the preservation of fertility.

Oncology (ASCO and ESMO) and gynecology (ESHRE and ASRM) societies see the freezing of semen, oocytes, and/or embryos as an effective option for preserving fertility. The preservation of ovarian and testicular tissue is considered an experimental technique and should not be routinely used, except in pre-pubescent children seen at reference research centers. The use of drugs such as gonadotropin-releasing hormone agonists (GnRHa) is not effective in ensuring fertility after cancer treatment, and therefore should not be used in fertility preservation protocols [unless there is insufficient time to initiate controlled ovarian stimulation (≤ 1 week)]. In the case of pelvic radiotherapy, ovarian transposition (surgical displacement of the ovary in the pelvis to a region not exposed to radiotherapy, a.k.a. oophoropexy) may be an option to optimize the fertility of cancer patients (Figure 3) (Loren *et al*., 2013; Peccatori *et al*., 2013; The Practice Committee of American Society for Reproductive Medicine, 2013).

### Protocols and duration of assisted reproduction cycles

Delayed start of cancer treatment is one of the main barriers to the referral of patients for fertility preservation. However, performing ART cycles in these cases does not change the timing of cancer treatment initiation, considering that chemotherapy is usually started two to three weeks after the patients have been diagnosed (patients have to undergo additional testing before the start of cancer treatment), which is more than enough to have eggs/embryos stored (Levine *et al*., 2010).

For men, fertility preservation can occur immediately after serological tests and the administration of prophylactic antibiotics. Depending on the initial spermogram, male patients might have to collect multiple semen samples so that proper quantities of sperm are stored (Loren *et al*., 2013; Pentheroudakis *et al*., 2010).

For women, the duration of ovulation induction with gonadotropins and/or selective estrogen receptor modulators (SERMs) and/or aromatase inhibitors is approximately 10-14 days. Since the time to complete the fertility preservation cycle in women has to be optimized, ovarian stimulation can be initiated at any stage of the menstrual cycle (“random start stimulation”) and, if possible and necessary, successive cycles can be performed to increase the number of stored eggs and to achieve pregnancy after the patient has been cured from cancer (Cakmak *et al.,* 2013; Tsampras *et al*., 2017; von Wolff *et al*., 2018). Starting ovarian stimulation in the luteal phase allows the collection of a sufficient number of eggs and the achievement of similar pregnancy rates compared to cycles started in the early follicular phase (Boots *et al*., 2016).

**Figure 4:**
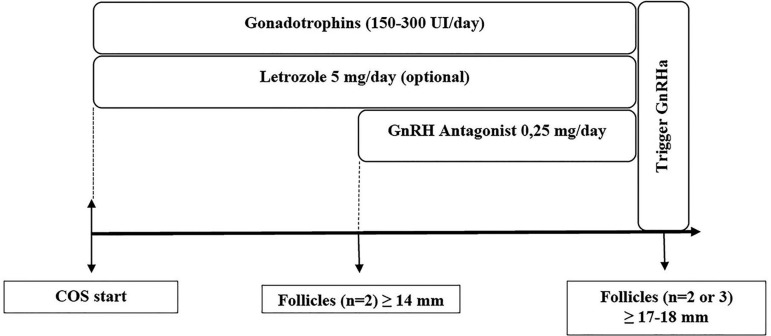
Start of controlled ovarian stimulation in the early follicular phase in women with cancer COS: controlled ovarian stimulation; GnRH: gonadotropin-releasing hormone; GnRHa: GnRH agonist

Controlled ovarian stimulation (COS) has its particularities depending on the phase of the menstrual cycle:

**A) Early Follicular Phase:** There are no differences in the COS protocols of women with and without cancer. Gonadotropins, selective estrogen receptor modulators (SERMs), and/or aromatase inhibitors (preferred in estrogen-dependent cancer cases) are initiated between the second and fourth day of the menstrual cycle. When two or more follicles are ≥ 14 mm in size, a gonadotropin-releasing hormone (GnRH) antagonist is added and maintained until there is indication for a GnRH agonist trigger (Figure 4). In order to obtain an adequate number of oocytes and enhance live birth rates, gonadotropin doses tend to be higher in fertility preservation cycles. Since patients may therefore be at higher risk of ovarian hyperstimulation, a GnRHa trigger is preferably used to minimize this effect (von Wolff *et al*., 2018). It is important to adequately evaluate the ovarian reserve considering the age of the woman, her antral follicle count on ultrasound examination, and hormone levels (AMH and FSH, if possible) (Goldrat *et al*., 2015).

**Figure 5:**
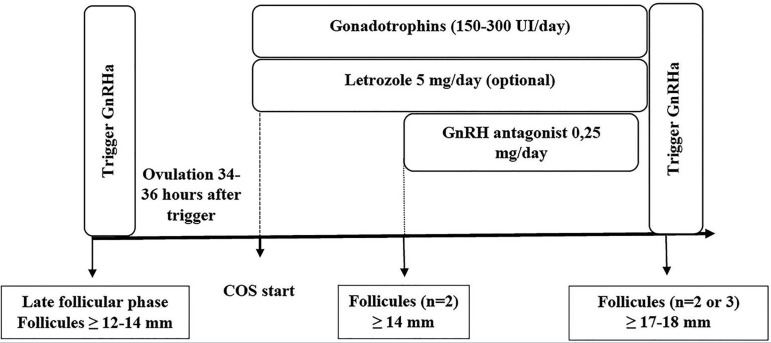
Start of controlled ovarian stimulation in the late follicular phase in women with cancer. COS: controlled ovarian stimulation; GnRH: gonadotropin-releasing hormone; GnRHa: GnRH agonist.

**B) Late follicular phase:** a GnRHa trigger can be administered when the follicles are larger than 12-14 mm. After ovulation (approximately 34-36 hours later), COS is started with gonadotropins and letrozole. Then, when two or more follicles measure ≥ 14 mm, the GnRH antagonist is added and maintained until the indication of the trigger with GnRHa (Figure 5) (Cakmak *et al*., 2013; von Wolff *et al.,* 2018).

**Figure 6:**
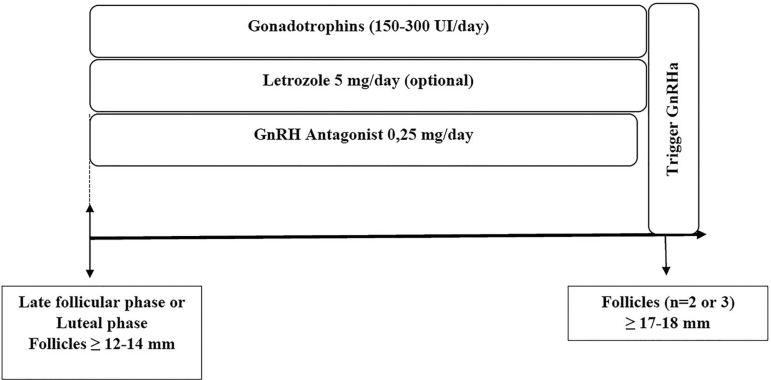
Start of controlled ovarian stimulation in the luteal phase in women with cancer (also an alternative when COS is started in the late follicular phase) COS: controlled ovarian stimulation; GnRH: gonadotropin-releasing hormone; GnRHa: GnRH agonist

**C) Luteal phase:** There may be recruitment of antral follicles during the luteal phase that precedes the menstrual cycle. Although only one follicle is selected as dominant around the fifth to ninth day of the menstrual cycle, some follicles recruited in the previous luteal phase may not undergo atresia, and may therefore be sensitive to hormonal stimulation with gonadotropins (Baerwald *et al*., 2003). In the presence of follicles greater than 12-14 mm, a GnRH antagonist can be initiated at the start of COS with letrozole and gonadotropins. In these cases, there may be a greater need for gonadotropins and GnRH antagonists (Cakmak *et al*., 2013; Goldrat *et al*., 2015). This scheme may also be used in women initiating COS on the late follicular phase (Figure 6) (Goldrat *et al.,* 2015). In the absence of dominant follicles, COS can be initiated without a GnRH antagonist, which is added later on when the follicles are larger than 14 mm (von Wolff *et al*., 2018).

In women with estrogen-dependent tumors (breast and endometrial cancer), the fear is that prognosis may worsen with the increase in endogenous estrogen levels resulting from ovarian stimulation with gonadotropins. To minimize this effect, it is recommended to potentiate the availability of meiosis II (MII) eggs and decrease the dose of exogenous gonadotropins. Additionally, patients with estrogen-dependent cancer should be given a combination of gonadotropins and aromatase inhibitors. However, a recent meta-analysis comprising studies with early breast cancer patients showed that letrozole was not associated with greater numbers of mature eggs (MII) or increased disease-free intervals when compared to women on fertility preservation protocols without aromatase inhibitors (Rodgers *et al*., 2017).

### Double stimulation

The live birth rate varies depending on female age and number of cryopreserved oocytes. In general, live birth rates range from 70-80% in women younger than 38 years with 15 to 20 cryopreserved mature oocytes. In women between the ages of 38 and 40, the rates are 65-75% when 25 to 30 MII oocytes are harvested for cryopreservation (Doyle *et al*., 2016). These numbers demonstrate that in order to explore the reproductive potential of fertility preservation, more than one ovarian stimulation cycle (double stimulation) is required to obtain adequate numbers of preserved MII oocytes (Tsampras *et al*., 2017; von Wolff *et al*., 2018).

**Figure 7:**
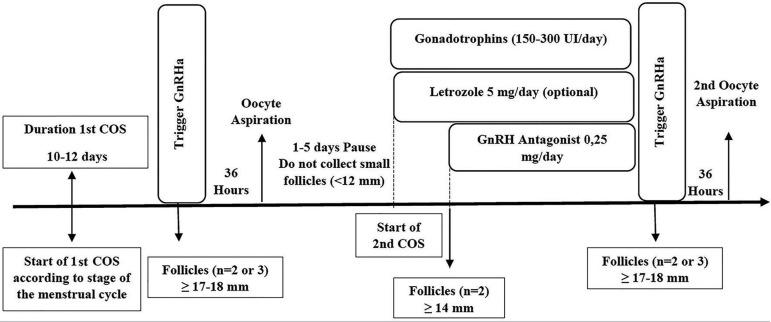
Double stimulation for fertility preservation in women with cancer COS: controlled ovarian stimulation; GnRH: gonadotropin-releasing hormone; GnRHa: GnRH agonist

In a double stimulation protocol, the use of gonadotropins can be initiated at any stage of the menstrual cycle (random start stimulation, as shown in Figures 4, 5, and 6), and in the first oocyte retrieval the antral follicles should not be aspirated. Between the first and fifth day after ovarian puncture, a new COS may be initiated and a GnRH antagonist administered when the dominant follicle is ≥14 mm. In general, double stimulation takes 24-30 days (Figure 7) (von Wolff *et al*., 2018). These cases require close interaction with the oncology team so that an adequate strategy is adopted to allow for fertility preservation without interfering with the cancer treatment schedule. Figure 2 shows a practical flowchart for fertility preservation.

### Contraception

In addition to the possible side effects of chemo and radiotherapy on patient reproductive potential, gonadal toxicity varies depending on the type of medication and dose of radiation prescribed (Table 1). Alkylating agents (nitrogen mustards, mechlorethamine, cyclophosphamide, ifosfamide, melphalan, chlorambucil); ethylene imines and methyl melamines (thiotepa and hexamethylmelamine); alkyl sulfonates (busulfan); nitrosureas (carmustine [BiCNU] and streptozotocin); triazenes (dacarbazine and temozolomide; platinum complexes (cisplatin, carboplatin, oxaliplatin); and subdiaphragmatic radiotherapy are the main types of cancer treatment that might harm fertility (Laurence & Rousset-Jablonski, 2012). Consequently, some women may present with variable/intermittent reproductive function and conceive during cancer treatment.

Physiologically, regular menstrual cycles associated with signs of ovulation (mucus, pain, periovulatory bleeding, premenstrual syndrome) suffice to predict ovulation (Practice Committee of American Society for Reproductive Medicine, 2012). However, the absence of menstruation or ovulation signs does not necessarily indicate that ovarian function is diminished in cancer survivors, since they may be able to conceive even when presenting amenorrhea (Lee *et al*., 2009).

In addition to a possibly unplanned pregnancy, women with cancer that become pregnant have higher mortality rates, shorter disease-free intervals (Hartman & Eslick, 2016), higher tumor growth rates, and higher chances of having heart failure, miscarriages, low birth weight newborns, and preterm newborns (Pentheroudakis *et al*., 2010).

Chemo and radiotherapy may also be associated with adverse gynecological symptoms (e.g.: irregular menstrual cycles) (American College of Obstetricians and Gynecologists, 2014) resulting from altered ovarian hormonal function, even when agents with low to intermediate risk of gonadal toxicity are used (Table 2) (Boran *et al*., 2005). In this scenario, contraceptives can offer additional benefits such as controlling abnormal uterine bleedings while providing women with cancer with higher levels of quality-of-life and optimizing their sex life by decreasing the fears of an unplanned pregnancy. All the factors cited above support the use of contraceptive counseling as a mandatory measure before the start of cancer treatment, since the impact of gonadal toxicity from cancer therapy cannot be predicted.

**Table 2:** Eligibility criteria for contraceptives in cases of breast, liver, cervical, endometrial, and ovarian cancer

Condition/Method	COC	CIC	Patch/Ring	POP	DMPA	Etonogestrel implants	Cu-IUD	LNG-IUD
**Breast **	3	3	3	3	3	3	1	3
**Liver **	4	3/4	4	3	3	3	1	3
**Cervical**	2	2	2	1	2	2	S:4/C:2	S:4/C:2
**Endometrial **	1	1	1	1	1	1	S:4/C:2	S:4/C:2
**Ovarian**	1	1	1	1	1	1	S:3/C:2	S:3/C:2

COC: combined oral contraceptives; CIC: monthly combined injectable contraceptives; POP: progestogen-only pill; DMPA: depot medroxyprogesterone acetate; Cu-IUD: copper intrauterine device; LNG-IUD: levonorgestrel-releasing intrauterine device; S: to start; C: to continue

### Choice of contraceptive method

The World Health Organization (WHO) and the Centers for Disease Control and Prevention (CDC) established medical eligibility criteria for contraceptives according to the presence of comorbidities. According to these guidelines, when a contraceptive method is included in category 1 or 2, it can be safely prescribed; when assigned to category 3, risks outweigh the benefits and the method should not be prescribed, unless there is no other alternative for the patient; category 4 indicates the method should never be prescribed due to unacceptable levels of risk to health. The main types of gynecological cancer were considered in these recommendations (World Health Organization, 2015; Curtis *et al*., 2016).

The WHO and the CDC established recommendations regarding the eligibility of contraceptives vis-à-vis the main gynecological tumor types, but not for other cancers affecting women of reproductive age such as lymphoma, leukemia, other less frequent tumors, or for use during chemo and radiotherapy. This gap might be a consequence of the lack of studies investigating contraception in these scenarios.

Women with cancer require effective long-term contraception. Therefore, they should be ideally prescribed long-term contraceptives for their greater effectiveness and higher compliance levels (Laurence & Rousset-Jablonski, 2012; Patel & Schwarz, 2012). However, it is important to consider safety before prescribing contraceptives, considering the CDC/WHO eligibility criteria and factors such as tumor type [estrogen-dependent (breast cancer, endometrial) *vs.* not], thrombogenic risk associated with the tumor and chemotherapy regimen, immunosuppression, presence of blood disorders (thrombocytopenia/anemia), and bone mass reduction, as well as possible metabolic, cardiovascular, and drug interactions between the chemotherapeutic agent and the contraceptive (metabolism mediated by cytochrome P450 3A4).

### Tumor type

In the WHO and CDC eligibility criteria, the use of contraceptive methods was considered for cases of cervical, breast, endometrial, ovarian, and liver cancer. Of these tumor types, breast (even if the patient has been considered cured after five years of treatment) and liver cancer (hepatocellular carcinoma) are contraindications for the use of hormonal contraceptives. In these situations, only copper intrauterine devices are eligible (Table 2) (World Health Organization, 2015; Curtis *et al*., 2016). Hormonal contraceptives are not contraindicated for patients with malignant endometrial, ovarian, or cervical tumors. However, there is a particularity concerning copper and levonorgestrel (LNG) intrauterine devices (IUD): if a patient is diagnosed with cervical, endometrial, or ovarian cancer while using an IUD, she can keep it. However, if a patient diagnosed with one of these conditions seeks gynecological care to have a prescription for contraceptives, she cannot be offered an IUD (Table 2) (World Health Organization, 2015; Curtis *et al*., 2016).

Few studies with adequate external validity have looked into contraception for other tumors. For neoplasms not affecting the liver and non-estrogen-dependent tumors, any hormonal contraceptive may be used as long as there is no contraindication for other reasons such as thrombosis, metabolic risk, etc. (Laurence & Rousset-Jablonski, 2012; Patel & Schwarz, 2012). In addition, the Society of Family Planning (SFP) recommends that combined hormonal methods should not be used by women with cancer on account of the risk of venous thromboembolism (VTE), as discussed below (Patel & Schwarz, 2012).

### Venous thromboembolism

In 2012, the SFP published a clinical guideline on the safety and efficacy of contraceptives for women with cancer. In its recommendations, the SFP suggested that women with malignant tumors should avoid the combination of hormonal contraceptives (regardless of route of administration) due to increased risk of VTE, since cancer (Khorana *et al*., 2013), estrogen, and possibly some chemotherapeutic agents are independent risk factors for venous thrombosis (Patel & Schwarz, 2012).

The isolated use of progestogen does not seems to be associated with the development of VTE, since the WHO and CDC deemed the use of these contraceptives to be safe in different situations associated with risk of thromboembolic events (World Health Organization, 2015; Curtis *et al*., 2016). According to the CDC and the WHO, isolated progestogens are safe for women with cervical, endometrial, and ovarian cancer (World Health Organization, 2015; Curtis *et al*., 2016). However, there are no studies on the safety of this class of contraceptives regarding the risk of VTE for other types of tumors.

Although alkylating agents might be associated with increased risk of VTE in women with breast cancer with a mean age of 60 years (Nolan *et al*., 2011), there are no studies looking into the risk of VTE of women of reproductive age. Given the lack of studies investigating the use of progestogens alone and the risk of VTE, these contraceptives can be used because of their high efficacy and non-contraceptive benefits.

### Blood disorders

Depending on the type of cancer, treatment schedule and duration, chemotherapy may cause anemia and severe thrombocytopenia. Patients with hematologic malignancies are at increased risk of bleeding for having decreased platelet counts (< 10,000) (Stanworth *et al*., 2015). For this reason, it is important not to use injectable medication, since intramuscular injections into the deltoid or gluteus muscle may favor the development of hematomas and abscesses (Laurence & Rousset-Jablonski, 2012). Methods that require manipulation of the uterus (copper and LNG IUDs) and etonogestrel contraceptive implants should also be avoided in these scenarios for risk of bleeding. This information can be inferred from the recommendations of the CDC and the WHO on the prescription of IUDs for women with severe thrombocytopenia and systemic lupus erythematosus, which state that IUDs should not be initiated but may be maintained if the device was put in place prior to the diagnosis of thrombocytopenia (World Health Organization, 2015; Curtis *et al*., 2016).

Cancer and chemotherapy might be associated with the development of anemia (Ray-Coquard *et al*., 2012). In these cases, the non-contraceptive benefits of these drugs should be explored through methods that induce a higher rate of amenorrhea. Progestogen-only methods are the best for bleeding control (amenorrhea or decreased bleeding duration and/or volume): 50-70% for the LNG-IUD and 46-90% with the quarterly injection (Hubacher *et al*., 2009). As recommended by the SFP, women with cancer who develop anemia can use any progestogen-only method (Patel & Schwarz, 2012), although there are no studies demonstrating safety against VTE.

Since the copper IUD might increase vaginal bleeding by 18-20% during the first three months of use, and in view of the severity of the clinical status of women with cancer concurrently with anemia and thrombocytopenia, copper IUDs should not be offered to individuals with severe anemia (although they can be left in place in women already using IUDs) (Patel & Schwarz, 2012).

### Osteoporosis

 The treatment of breast cancer and other estrogen-dependent tumors promotes a state of hypoestrogenism that contributes to bone mass reduction and increases the risk of developing osteoporosis/fractures throughout life (Milat & Vincent, 2015). Among contraceptives, the depot medroxyprogesterone acetate (DMPA) quarterly injection may be associated with a transient reduction in bone mineral density (Gai *et al*., 2011), a trait that might be detrimental to groups at risk of osteoporosis (women with cancer, for example).

Cancer patients presenting other risk factors for osteoporosis should be offered diets with adequate levels of calcium, perform higher-impact physical exercises (if possible), and discuss whether progestogen-only contraception is indeed necessary in reference to other significant objectives, such as the induction of amenorrhea. In situations where the maximum acquisition of bone mineral density (adolescence) has not yet occurred, the CDC, the WHO, and the Society for Adolescent Medicine (World Health Organization, 2015; Curtis *et al*., 2016) support the use of DMPA (Level A), but there is no information on the use of progestogen-only contraceptives in cancer patients. According to the SFP, DPMA should be avoided in women who develop osteopenia and osteoporosis after chemotherapy (Patel & Schwarz, 2012).

### Metabolic and cardiovascular disorders

Cardiac complications may be a side effect of thoracic radiotherapy and some chemotherapy agents, such as anthracyclines (daunorubicin, doxorubicin, epirubicin, idarubicin, mitoxantrone, pixantrone, valrubicin), trastuzumab, taxanes (paclitaxel), angiogenesis inhibitors, 5-fluorouracil (5-FU), and capecitabine (arterial coronary disease) (Khawaja *et al*., 2014). Cardiac toxicity has been associated with patient age at the time of diagnosis, cumulative dose of chemotherapy, radiation dose, and black race, among other factors (Lipshultz *et al*., 2014).

Since women with cancer are at higher risk for VTE and in view of the possible cardiac toxicity of some cancer treatments, the prescription of combined hormonal contraceptives should be avoided. The eligibility of other contraceptive methods for women with cancer should be assessed according to the guidelines of the WHO (World Health Organization, 2015) and the CDC (Curtis *et al*., 2016). According to these guidelines, women with multiple risk factors for coronary artery disease should not be prescribed combined methods or depot medroxyprogesterone acetate (DMPA). DMPA has been associated with decreased HDL levels, which limits its use in situations of cardiovascular risk (Table 3) (World Health Organization, 2015; Curtis *et al*., 2016). Patient with other metabolic and cardiovascular conditions should follow the CDC and the WHO guidelines, although there are no studies on the metabolic and cardiovascular safety of contraceptives in women of reproductive age with cancer.

**Table 3:** Eligibility criteria for contraceptives in cases of multiple risk factors for cardiovascular disease

Condition/ Method	COC	CIC	Patch/Ring	POP	DMPA	Etonogestrel implants	Cu-IUD	LNG-IUD
**Multiple risk factors for CAD**	3/4	3/4	3/4	2	3	2	1	1

Note: Adapted from the Medical Eligibility Criteria for Contraceptive Use, 5^th^ ed.,2015. COC: combined oral contraceptives; CIC: monthly combined injectable contraceptives; POP: progestogen-only pill; DMPA: depot medroxyprogesterone acetate; Cu-IUD: copper intrauterine device; LNG-IUD: levonorgestrel-releasing intrauterine device; S: to start; C: to continue

### Immunosuppression

The information related to contraceptives and immunosuppression is based on cases of solid tumor transplantation, systemic lupus erythematosus/rheumatoid arthritis, and human immunodeficiency virus (HIV) infection (with or without AIDS) (World Health Organization, 2015; Curtis *et al*., 2016).

According to the CDC, female recipients of solid organ transplants can be prescribed hormonal contraceptives and copper IUDs. However, in the presence of complications (graft failure, rejection, cardiac allograft vasculopathy), the combined methods should not be used, while progestogen-only contraceptives become eligible. In these cases, LNG-IUD and Cu-IUD should not be offered, but patients with IUDs in place prior to transplantation can keep them (Curtis *et al*., 2016).

In the case of immunosuppressive treatment of patients with systemic lupus erythematosus/rheumatoid arthritis, any contraceptive method is eligible, including IUDs (the CDC does not recommend DMPA to individuals on chronic glucocorticoids for the treatment of rheumatoid arthritis). According to the CDC, immunosuppressed women with HIV infection (CD4 lower than 200) may also use any contraceptive method (Curtis *et al*., 2016). However, the WHO does not recommend IUDs in these cases (although they can be kept if the patient had been using an IUD before being diagnosed with HIV infection) (World Health Organization, 2015).

Regarding secondary immunosuppression following hematopoietic cell transplantation, there is a report of a woman who kept her LNG-IUD during chemotherapy without developing infection or irregular vaginal bleeding, even in the presence of neutropenia and thrombocytopenia (Brady *et al*., 2017). So far, no studies have looked into women immunosuppressed by cancer treatment using IUDs. Thus, immunosuppressed women with cancer may receive progestogen-only methods. LNG-IUD and Cu-IUD should not be initiated but may be kept if the patient had already been using an IUD prior to transplantation.

### Drug interaction

Many steroidal hormones and some medications (anticonvulsants, for example) are substrates for the cytochrome P450 enzyme system, particularly the 3A4 isoenzyme. Therefore, some contraceptives (oral progestogen-only and combined hormones) may have reduced efficacy and even decrease the effectiveness of other drugs because of the hepatic first-pass effect mediated by cytochrome P450 3A4 (Dutton & Foldvary-Schaefer, 2008). There are no studies on the pharmacokinetics of contraceptives in women with cancer, but some chemotherapeutics may interfere with the cytochrome P450-mediated metabolism (Sweiss *et al*., 2019), which suggests that the effectiveness of contraceptives/chemotherapy might be altered. Figure 2 presents a practical flowchart for the holistic approach to infertility: preservation and contraception.

## CONCLUSION

The fertility approach for women with cancer should include counseling on fertility preservation techniques and contraception options. Oocyte/embryo cryopreservation are effective techniques and the procedure does not delay the start of cancer treatment, since COS can be initiated at any stage of the menstrual cycle. At the same time, contraceptive counseling should be conducted on the basis of the CDC/WHO eligibility criteria. However, there are few studies on the safety of contraceptives in cancer patients, and before choosing the contraceptive method, other aspects should be considered, such as the type of tumor, thrombogenic risk associated with cancer or chemotherapy, immunosuppression, blood disorders (thrombocytopenia/anemia), and reduction of bone mass, in addition to the possible metabolic, cardiovascular, and drug interactions between chemotherapeutic agents and contraceptives.
